# Susceptibility to particle health effects, miRNA and exosomes: rationale and study protocol of the SPHERE study

**DOI:** 10.1186/1471-2458-14-1137

**Published:** 2014-11-04

**Authors:** Valentina Bollati, Simona Iodice, Chiara Favero, Laura Angelici, Benedetta Albetti, Raquel Cacace, Laura Cantone, Michele Carugno, Tommaso Cavalleri, Barbara De Giorgio, Laura Dioni, Silvia Fustinoni, Mirjam Hoxha, Barbara Marinelli, Valeria Motta, Lorenzo Patrini, Laura Pergoli, Luciano Riboldi, Giovanna Rizzo, Federica Rota, Sabrina Sucato, Letizia Tarantini, Amedea Silvia Tirelli, Luisella Vigna, Pieralberto Bertazzi, Angela Cecilia Pesatori

**Affiliations:** Molecular Epidemiology and Environmental Epigenetics Laboratory, Department of Clinical Sciences and Community Health, Università degli Studi di Milano, Milan, Italy; Epidemiology Unit, Fondazione IRCCS Ca’ Granda Ospedale Maggiore Policlinico, Milan, Italy; Laboratory of Clinical Chemistry & Microbiology, Fondazione IRCCS Ca’ Granda Ospedale Maggiore Policlinico, Milan, Italy; Worker’s Health Protection and Promotion Unit, Fondazione IRCCS Ca’ Granda Ospedale Maggiore Policlinico, Milan, Italy

**Keywords:** Particulate matter, Obesity, Cardiovascular effects, Extracellular vesicles, Exosomes, Microvesicles, miRNAs

## Abstract

**Background:**

Despite epidemiological findings showing increased air pollution related cardiovascular diseases (CVD), the knowledge of the involved molecular mechanisms remains moderate or weak. Particulate matter (PM) produces a local strong inflammatory reaction in the pulmonary environment but there is no final evidence that PM physically enters and deposits in blood vessels. Extracellular vesicles (EVs) and their miRNA cargo might be the ideal candidate to mediate the effects of PM, since they could be potentially produced by the respiratory system, reach the systemic circulation and lead to the development of cardiovascular effects.

The SPHERE (“Susceptibility to Particle Health Effects, miRNAs and Exosomes”) project was granted by ERC-2011-StG 282413, to examine possible molecular mechanisms underlying the effects of PM exposure in relation to health outcomes.

**Methods/design:**

The study population will include 2000 overweight (25 < BMI < 30 kg/cm^2^) or obese (BMI ≥ 30 kg/cm^2^) subjects presenting at the Center for Obesity and Work (Fondazione IRCCS Ca’ Granda Ospedale Maggiore Policlinico, Milan, Italy).

Each subject donates blood, urine and hair samples. Extensive epidemiological and clinical data are collected. Exposure to PM is assigned to each subject using both daily PM_10_ concentration series from air quality monitors and pollutant levels estimated by the FARM (Flexible air Quality Regional Model) modelling system and elaborated by the Regional Environmental Protection Agency.

The recruitment period started in September 2010 and will continue until 2015. At December 31, 2013 we recruited 1250 subjects, of whom 87% lived in the province of Milan.

Primary study outcomes include cardiometabolic and respiratory health effects. The main molecular mechanism we are investigating focuses on EV-associated microRNAs.

**Discussion:**

SPHERE is the first large study aimed to explore EVs as a novel potential mechanism of how air pollution exposure acts in a highly susceptible population. The rigorous study design, the availability of banked biological samples and the potential to integrate epidemiological, clinical and molecular data will also furnish a powerful base for investigating different complementary molecular mechanisms. Our findings, if confirmed, could lead to the identification of potentially reversible alterations that might be considered as possible targets for new diagnostic and therapeutic interventions.

**Electronic supplementary material:**

The online version of this article (doi:10.1186/1471-2458-14-1137) contains supplementary material, which is available to authorized users.

## Background

Air pollution is a major health concern which accounts for ~3.7 million global deaths annually, according to World Health Organization (WHO) estimates [1].

Numerous health studies have shown acute [2–7] and chronic [8–10] particulate air pollution exposures to be associated with early death, particularly from cardiovascular and respiratory diseases [3, 10, 11]. Metals, which are constituents of particulate air pollution, have been shown to be associated with cardiovascular diseases (CVD) as well [12–31]. Epidemiological and animal studies have suggested many potential mechanisms by which particles may impact health. Airway or parenchymal inflammatory responses to particulate matter (PM) have been hypothesized to be the inciting events of a cascade of pathophysiologic changes in autonomic cardiac, systemic inflammatory, and haemostatic activities. All these processes may ultimately lead to the acute events associated with PM exposure [32].

One of the most important gaps in our current knowledge regarding PM-related health effects is the identification of susceptible subjects [33]. Recent research findings pointed out obesity as a susceptibility factor to the adverse effects of PM exposure partly due to an increase in particle absorption [34]. A positive correlation between exhaled nitric oxide, a marker of pulmonary inflammation, and Body Mass Index (BMI) has been shown in healthy adults [35]. BMI was associated with a graded increase in the estimated total lung dose of deposited fine particles in an inhalation study of healthy children (6–13 years of age) [36]. In a panel study of 44 senior citizens, vascular inflammatory response (measured by C-reactive protein) to ambient levels of PM_2.5_ (particulate matter with aerodynamic diameter ≤2.5 μm) averaged over 1–7 days was greater in obese (BMI ≥30 kg/m^2^) than in non-obese subjects [37]. Moreover, a differential autonomic cardiac response (measured as heart rate variability) to metal particulates has been observed between obese and non-obese individuals [33].

The mechanisms linking PM exposure and CVD have not yet been fully elucidated. It has been proposed that inhaled fine particulate matter translocates directly into the systemic circulation through the pulmonary capillary bed, promoting atherothrombosis by breaching endothelial integrity and inciting a local inflammatory reaction [38]. However, just a very small fraction of these fine and ultrafine particles accumulate in extra pulmonary organs such as the liver and the spleen, [39] and currently there is no final evidence that fine particles physically enter and deposit in blood vessels. An alternative hypothesis is that ambient particles produce a strong inflammatory reaction in the lungs followed by the release of pro-inflammatory mediators that are able to reach the systemic circulation [40, 41]. Despite more than two decades of mechanistic research, the recent statement of air pollution and cardiovascular disease from the American Heart Association remarked that compared to the high degree of consistency of the epidemiological findings showing increased cardiovascular risk, the evidence on intermediate mechanisms remains moderate or weak [42].

Beside the release of pro-inflammatory mediators, cell-derived membrane vesicles are also released, representing another way of intercellular communication that has recently become the subject of increasing interest [41]. Extracellular vesicles (EVs) are spherical structures limited by a lipid bilayer that can be generated by cells and secreted into the extracellular space and are likely composed of both exosomes (EXs) and microvesicles (MVs). The term exosome is used to identify a particular subgroup of vesicles, ranging from 40 to 100 nanometers (nm) in diameter, released as a consequence of multivesicular endosome (a membrane-bound intracellular vesicle, containing EXs) fusion with the plasma membrane, [43] whereas the term microvesicle is used for those EVs, larger than 100 nm in diameter, that are shed from the plasma membrane.

EVs are released from cell membranes as a consequence of triggers such as endotoxin encounter, hypoxia or oxidative stress conditions, cytokines release, thrombin production [44] and could be one of the means used by tissues to adapt to these stimuli [45]. EV membranes are enriched in specific surface molecules which could favor their capture by recipient cells. The fate of EVs, after binding the surface of recipient cells, is not known but recent evidences suggest that they might fuse and deliver their content directly into the cytoplasm. In addition, after internalization in the target cells through surface-expressed ligands, EVs may transfer microRNAs (miRNAs) [46, 47] allowing intercellular and inter-organ communication in the body [47]. MiRNAs are small, endogenous, single stranded noncoding RNAs of 20–22 nucleotides [48] that post-transcriptionally regulate gene expression by either triggering mRNA cleavage or repressing translation [49]. One single miRNA can regulate hundreds of mRNAs in interrelated gene pathways and a single mRNA can be targeted by several different miRNAs [50]. Moreover, miRNA expression in circulating EVs has been detected in plasma of normal subjects and a predictive role of peripheral blood miRNA signatures in human diseases has also been hypothesized [47].

### Aims and hypotheses

**Figure 1:**
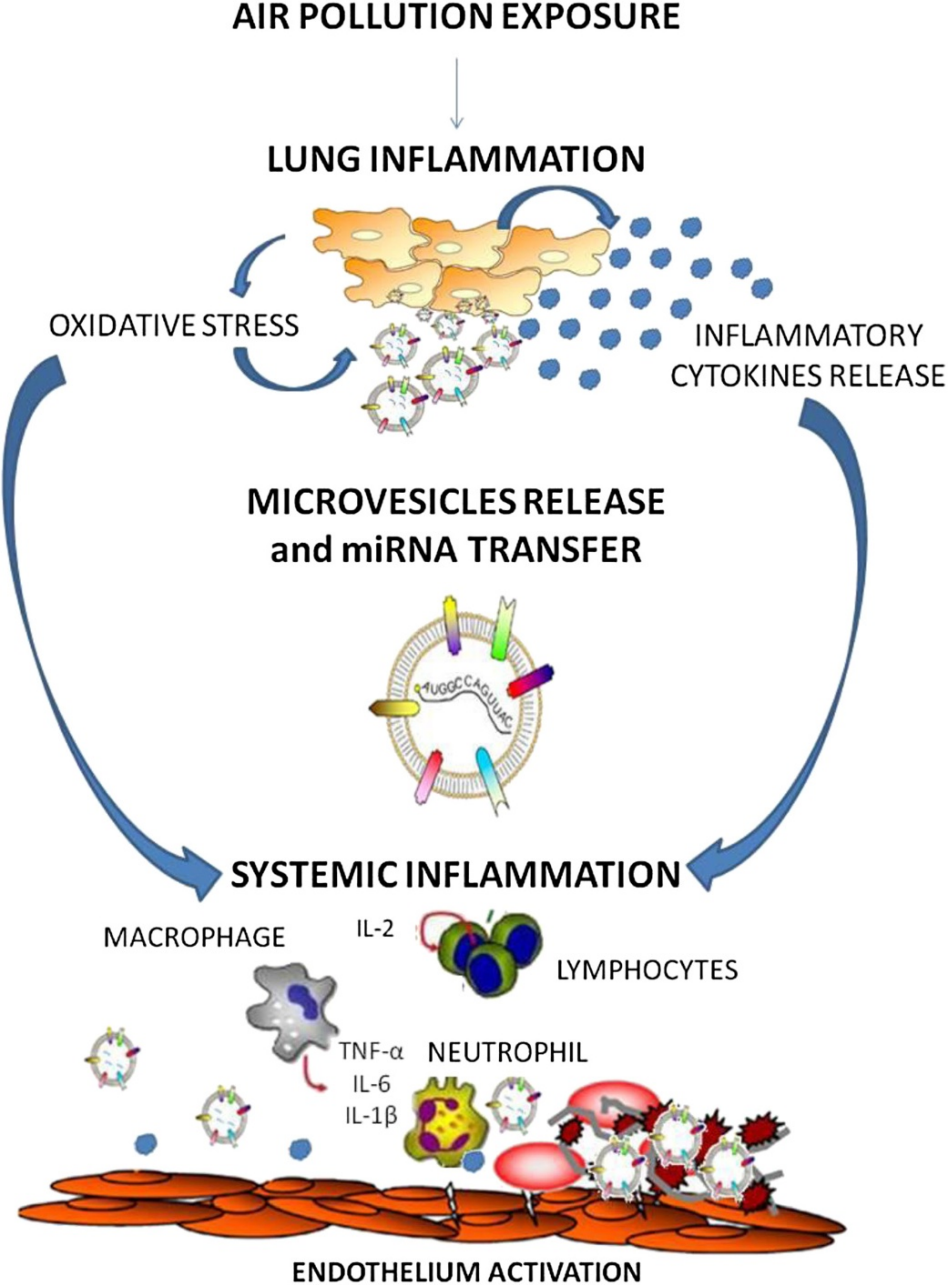
**Proposed mechanism for air pollution effects on microvesicle release and cell-to-cell communication.**

Taken together (Figure 1), these findings suggest that:

– Obese individuals might represent one of the best population to investigate the effects of environmental air particles on several molecular mechanisms and, as a final objective, on cardiovascular and respiratory parameters;
– EVs might be the ideal candidate mechanism to mediate the effects of air pollution, since they could potentially be produced by the respiratory system, [51, 52] reach the systemic circulation [53] and lead to the development of endothelial dysfunctions [54].

**Figure 2:**
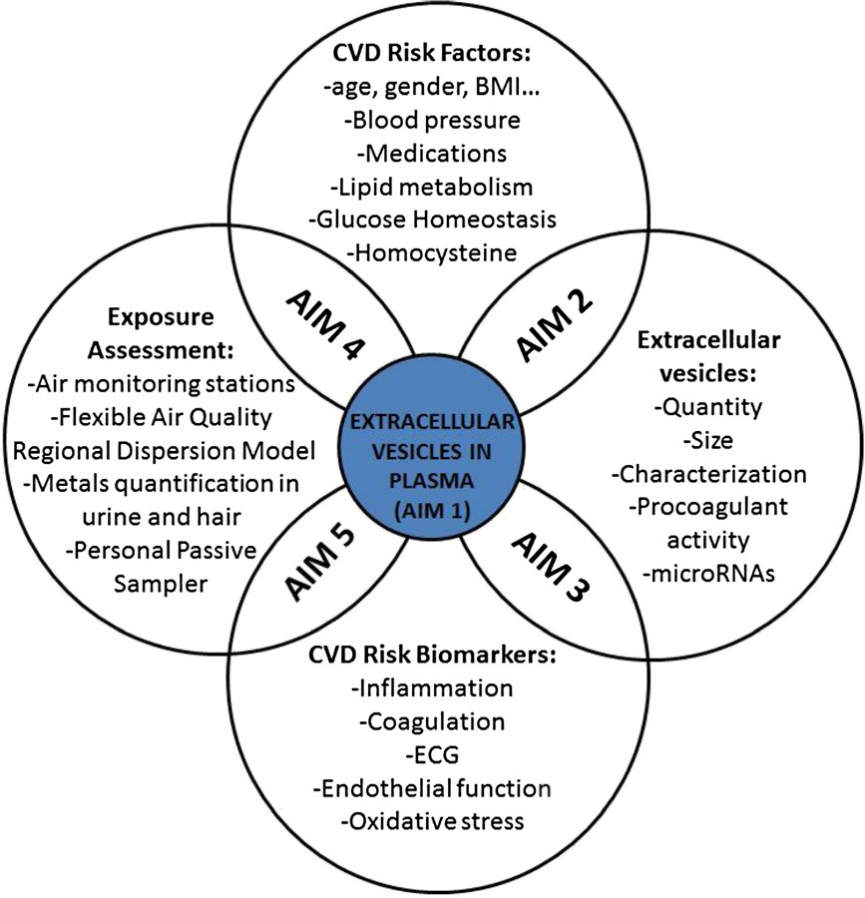
**Conceptual model for the SPHERE study.**

The specific aims of the first funded SPHERE grant (ERC-2011-StG 282413 to Valentina Bollati), are summarized in Figure 2 and reported below:

1. Determine whether exposure to air particles and PM-associated metals can modify EVs in plasma as quantity, size, characterization of the membrane molecules, procoagulant activity, miRNA content.
2. Determine whether the changes found in EVs are associated with CVD risk factors, such as aging, gender, smoking habits, alcohol consumption, BMI, blood pressure, medications, lipid metabolism, glucose homeostasis, homocysteine.
3. Determine whether the changes found in EVs predict alterations of CVD risk endpoints, such as inflammation markers (Cytokines), ECG (QRS, PR waves and heart rate), endothelial function (ICAM-1, VCAM-1, C-reactive protein), oxidative stress (8-hydroxy-2′-deoxyguanosine).
4. Determine whether exposure to air particles and PM-associated metals are associated with selected CVD risk factors (listed in aim 2).
5. Determine whether exposure to air particles and PM-associated metals predict alterations of CVD risk biomarkers (listed in 3).

We hereby present study design, field activities, management organization and characteristics of the enrolled subjects.

## Methods/design

### Study design

The SPHERE study is a cross-sectional study investigating the effects of particulate air pollution on a population of susceptible overweight/obese subjects, residing in Lombardy Region, Italy.

Lombardy is situated in the Northern part of Italy and is physically divided into three parts from north to south: Alpine and pre-Alpine mountains, foothills, and a zone of alluvial plains sloping to the Po river. The region covers an area of 23.864 km^2^ with a population of about 10 million people [55] and consists of 12 provinces, among which Milan is the regional capital. The Milan metropolitan area counts 7 million inhabitants with 1.3 million residing in the core municipality [56].

### Study subjects

The overall study enrollment target is to recruit 2,000 participants presenting at the Center for Obesity and Work, Fondazione IRCCS Ca’ Granda Ospedale Maggiore Policlinico, Milan, Italy. The recruitment period started in September 2010 and will continue until the end of 2015. At December 31, 2013 we recruited 1250 subjects, 87% of whom living in the province of Milan. The eligibility criteria for participants are: 1) older than 18 years at enrollment; 2) obese/overweight according to the following definition: overweight is defined as a BMI between 25 and 30 kg/cm^2^, obesity is defined as a BMI of 30 kg/cm^2^ or more; 3) resident in Lombardy at the time of the recruitment; 4) agreement to sign an informed consent and donate blood and urine samples.

Exclusion criteria include: previous diagnosis of cancer, heart disease or stroke in the last year or other chronic diseases such as multiple sclerosis, Alzheimer’s disease, Parkinson’s disease, depression, bipolar disorder, schizophrenia and epilepsy [57]. The participation rate in the years 2010–2013 was 90%. The main characteristics of study participants are reported in Tables 1 and 2. Most participants are females (73.6%). The mean BMI is 33.5 Kg/cm^2^ (±5.5): 27.8% are overweight, 38.6% obese, and 33.6% severe obese (BMI ≥35 Kg/cm^2^). The mean age is 51.9 years (±13.6).

**Table 1:** **Demographic and lifestyle characteristics of study participants at 31/12/2013**

Characteristics	Categories	n = 1250
Sex	Male	330 (26.4%)
	Female	920 (73.6%)
Age	Years (mean ± SD)	51.9 ± 13.6
Education	Primary school or less	105 (8.4%)
	Secondary school	325 (26.0%)
	High school	493 (39.4%)
	University	188 (15.0%)
	Others	87 (7.0%)
	Missing	52 (4.2%)
Occupation	Employee	714 (57.1%)
	Unemployed	102 (8.2%)
	Pensioner	304 (24.3%)
	Housewife	93 (7.4%)
	Missing	37 (3.0%)
Ethnicity	White	1198(95.8%)
	Black	11 (0.9%)
	Asian	3 (0.3%)
	South America	38 (3.0%)
Year of enrollment	2010	129 (10.3%)
	2011	419 (33.5%)
	2012	385 (30.8%)
	2013	317 (25.4%)
Season of enrollment	Winter	320 (25.6%)
	Spring	313 (25.0%)
	Summer	190 (15.2%)
	Autumn	427 (34.2%)
Smoking	Never	599 (47.9%)
	Former	431 (34.5%)
	Current	190 (15.2%)
	Missing	30 (2.4%)
Cigarettes smoked* [cigarettes/day]	<= 5	53 (27.9%)
	5-10	53 (27.9%)
	10-15	33 (17.4%)
	15-20	37 (19.6%)
	20-40	13 (6.8%)
	Missing	1 (0.5%)
Time since quitting (n = 419)	Median [Q1, Q3]	13.1 [5.8–23.4]
Pack/years (n = 1153)	Median [Q1, Q3]	
Among current and former smokers		14.5 [6.1–28.0]
Including nonsmokers		0 [0–13.5]
Alcohol consumption	Yes	636 (50.9%)
	No	518 (41.4%)
	Missing	96 (7.7%)
Residence area	City	534 (42.7%)
	Peripheral area	331 (26.5%)
	Rural area	30 (2.4%)
	Village/small city	206 (16.5%)
	Missing	149 (11.9%)
Living area	Province of Milan (Excluding City of Milan)	379 (30.3%)
	*City of Milan*	713 (57.0%)
	Outside Milan	158 (12.7%)
Work area	Province of Milan (Excluding City of Milan)	94 (13.1%)
	*City of Milan*	339 (47.5%)
	Outside Milan	34 (4.8%)
	Missing	247 (34.6%)
Floor of residence	Ground floor	223 (17.8%)
	First floor	244 (19.5%)
	Second floor	156 (12.5%)
	Beyond second floor	471 (37.7%)
	Missing	156 (12.5.%)
Residence traffic exposure	Mild	108 (8.7%)
	Moderate	595 (47.6%)
	Heavy	369 (29.5%)
	Missing	178 (14.2%)

*Among current smokers; Q1: first quartile; Q3: third quartile.

**Table 2:** **Main clinical characteristics of the study subjects at December 31, 2013**

Characteristics	N	
BMI, Kg/cm^2^	1247	33.5 ± 5.5
BMI categorical		
<30 Kg/cm^2^		347 (27.8%)
30-35 Kg/cm^2^		483 (38.6%)
≥35 Kg/cm^2^		420 (33.6%)
Waist circumference, cm	1237	101.3 ± 13.1
Blood pressure, mmHg	1247	
Sistolic		125.4 ± 15.8
Diastolic		78.5 ± 9.5
Above 140/90 mmHg		60 (4.8%)
Below 140/90 mmHg		1190 (95.2%)
Heart rate, bpm	1243	67.6 ± 10.4
Uric acid	1163	5.2 ± 1.4
Fibrinogen, mg/dl	1129	335 ± 59
C-reactive protein	1160	0.3 [0.1-0.5]
Total cholesterol, mg/dl	1165	215.1 ± 41
HDL		59.2 ± 15.5
LDL		134.7 ± 36.3
Triglyceride	1164	107 [77–145.5]
Serum creatinine, mg/dL	1165	0.8 ± 0.3
AST, U/I	1159	19 [16–23]
ALT, U/I	1160	21 [16–30.5]
Gamma-Glutamyltransferase, IU/L	1162	19 [13–30]
Glucose	1155	92 [86–101]
Homocysteine	1151	10.4 [8.6–12.7]
TSH	1163	1.7 [1.2–2.5]
Glycated hemoglobin, mmol/mol	1159	39 [36.6–43]
Postprandial glycaemia, mg/dl	1162	99 [90–112]
Insulin level	1158	12.3 [8.8–18]
2-hour post glucose insulin level	1155	46.4 [27.6–73]
Urinary pH	1144	5.6 ± 0.7
Emocrome	1156	
White blood cells		6.8 ± 1.7
Red blood cells		4.8 ± 0.4
Hemoglobin		13.8 ± 1.4
Hematocrit		40.7 ± 3.4
Mean Corpuscolar Volume		85.1 ± 6.4
Platelets		249.7 ± 59

Continuous variable are expressed as mean ± standard deviation (SD) or as median [first quartile-third quartile] if not normally distributed; discrete variables are expressed as counts (%).

### Epidemiological and clinical data collection

At recruitment, each study subject is asked to: v

– fill in a lifestyle and a diet questionnaire,
– donate a 15 ml blood sample (for molecular tests),
– provide a 50 ml urine sample (for metal internal dose assessment),
– provide a lock of hair cut next to the root in the occipital area of the head (for metal internal dose assessment).

As part of the routine protocol, for each subject presenting at the Center, physical examination (including weight and height measured by a nurse following standardized procedures), spirometry, and electrocardiogram (ECG) are performed and biochemical parameters are also collected, including Emocrome, Fibrinogen, C-reactive protein, Total cholesterol, HDL, LDL, Triglyceride, Serum creatinine, AST, ALT, Gamma-Glutamyltransferase, fasting blood glucose, Homocysteine, TSH, Glycated haemoglobin, Postprandial glycaemia, Insulin level, 2-hour post glucose insulin level, Urinary pH, Uric acid.

### Lifestyle questionnaire

The lifestyle questionnaire collects information on socio-demographic data, residential area (complete address, characteristics of the house, and traffic), education, smoking history including passive smoking at home and at workplace, past and present health status of both the subjects and their first-degree relatives, medications in the last year, employment history and address of the plant of their current work (currently employed subjects only), physical activity levels and sedentary behavior, commuting time and transport mode.

### Diet questionnaire

The questionnaire on dietary habits includes questions on the number of servings from each food in a usual week or month. Several different types of food are investigated, including: legumes, vegetables, fruits, nuts, red and white meat, fish, eggs, dairy products, cereals, snacks, oil and butter, alcoholic beverage, tea and coffee. Number of servings from each food are translated into usual daily micronutrients intake weighting for serving size, age class and gender.

Both questionnaires are checked for completeness at the time of data collection in order to ensure high quality data.

### Lung and cardiac function

Pulmonary functions are measured, at the same day of blood drawing, with an electronic flow volume spirometer V-max 22 with Autobox (SensorMedics), according to European Respiratory Society/American Thoracic Society guidelines (ERS/ATS 2005) [58]. Tests are performed on patients in the sitting position, and are repeated until at least three reproducible forced expiratory curves have been obtained. Lung function parameters are: forced expiratory volume in one second (FEV_1_),; forced vital capacity (FVC); best peak expiratory flow (PEF); forced expiratory flows at 25%, 50%, and 75% of FVC (FEF_25_, FEF_50_, FEF_75_); mid-expiratory flow (FEF_25–75_) derived from the best maneuver (defined as the one with the highest sum of FEV_1_ + FVC). The single breath carbon monoxide diffusing capacity (DLCO) is also measured [59]. All parameters are expressed as a percentage of the predicted normal values, [60] and adjusted for sex, age, height.

A resting ECG and rhythm strip is also recorded and blood pressure is measured with the participant supine, after 5 minutes of rest.

### Biological sample collection

Specific laboratory Standard Operating Procedures have been developed to ensure quality control of every step involved in biospecimen collection and storage.

Blood is collected using two EDTA tubes (7 ml) and one PAX tube. Blood samples are transported from the Center for Obesity and Work to the Molecular Epidemiology and Environmental Epigenetics laboratory (University of Milan) within 2 hours from the phlebotomy. EDTA blood is processed to obtain whole blood, buffy coat, plasma, DNA, EV pellet. EDTA-blood is centrifuged 1,100 × g for 15 minutes at room temperature to obtain platelet-free blood plasma and buffy coat. An aliquot of plasma is further centrifuged at 1,000, 2,000, and 3,000 × g for 15 minutes at 4°C to remove cell debris. To prepare EV pellet, 1.5 mL of fresh plasma is transferred into a ultracentrifuge tube (Quick-Seal®- Round-Top, Polypropylene, 13.5 mL-Beckman Coulter, Inc) and filled up with PBS, filtered with 0.10 μm pore size membrane (StericupR-VP, 0.10 μm, polyethersulfone filter- Merck Millipore) to minimalize the background contribution of interfering particles.

PAX tubes are used for RNA collection and extraction since they contain a solution that inhibits RNA degradation and gene induction as blood is drawn into the tube.

A 50 ml urine sample is also collected and frozen at -20°C.

A lock of hair is cut next to the root in the occipital area of the head. A segment of 3 cm length is then washed to remove external contaminations and dissolved in alkaline solvent.

Biospecimens are tracked through a secure database that stores detailed information on sample description, aliquoting and freezer locations.

Approximately 90% of study subjects donated a blood sample, 99% donated a urine sample and 57% donated a lock of hair.

### Extracellular vesicles and miRNA investigation

In the SPHERE project, we apply a novel methodology aimed at investigating EVs in human plasma. This comprehensive approach, involves the characterization of microvesicular membrane determinants (to assess their cellular origin) by Flow Cytometry, microvesicle size and count by Nanosight (nanoparticle tracking analysis, NTA), and evaluation of microvesicle content (miRNAs).

For miRNA analysis, we follow a two-stage, split sample study design. The first (discovery) stage involves genome-wide miRNA expression profiling among 1,000 participants (the first 1,000 subjects consecutively recruited). The second (validation) stage involves a replication analysis of the top 50 miRNAs that resulted from the first stage. In particular, Stage 1 will involve the use of OpenArray technology (Applied Bioscience) that allows to run 2,688 TaqMan® qPCR reactions in parallel. In Stage 2, we will replicate the results obtained on the discovery set by standard real time PCR on an Applied Biosystems 7900HT Real-Time PCR System. Given the nature of this study and the lack of prior information on the investigated association, a formal power calculation was not possible. A simulation study showed that a sample size of 1000 subjects allows to obtain a power of 98% to detect a modest effect (at least 0.1 SD change) on miRNAs expression, for a 1 SD change in exposure.

### Exposure assessment

Exposure is defined using a multifaceted approach, which integrates information on particulate air pollution exposure with personal dose of metals in urine and hair. In particular, PM_10_ is assigned to each subjects following two approaches: (1) daily PM_10_ concentration series from air quality monitors; (2) daily PM_10_ concentrations estimated with the FARM (Flexible Air quality Regional Model) model, a three-dimensional Eulerian grid model for dispersion, transformation and deposition of particulates, capable to simulate PM_10_ concentration using a 4 km–dispersion grid [7].

In addition, we will perform personal air pollution measures of PM (including also PM_2.5_), using passive samplers, on a subgroup of selected subjects (n = 200).

#### Air monitoring stations

We obtained from the Regional Environmental Protection Agency (ARPA Lombardia) recordings of daily air levels of PM_10_ measured from monitoring stations located at 154 different sites throughout Lombardy since 2001. Three of them are located in the city of Milan (“Verziere”, “Pascal-Città Studi” and “Senato”). Nitrogen dioxide (NO_2_), carbon monoxide (CO) and ozone (O_3_) measurements were also available. We used daily concentrations measured by single monitors in the study area to characterize PM_10_ exposure at the date of recruitment and back to 365 days before enrollment, for each subject. Thus, we were able to evaluate both short- and long-term exposure to the investigated pollutant.

We geocoded the addresses of monitoring stations and study subjects in order to assign to each subject the daily PM_10_ concentration from: (1) the nearest monitor to home address, defined as “subjects’ residence”; (2) the nearest monitor to the Center for Obesity and Work (defined as “Policlinico”; (3) the Milan mean daily exposure (averaging the three available city monitors), defined as “average Milan”.

In case of incomplete series, each missing value was imputed by using an algorithm that integrates the annual average of the incomplete series and PM_10_ concentrations of the nearest and more correlated monitors [61].

About 57% of SPHERE subjects lives in Milan and an additional 28% works in the city (even if they lived outside the city); overall 67% of subjects spent many hours a day in the city or travelling from workplace to residence.

We considered subject PM_10_ levels measured at the “Policlinico” station as representative of mean PM_10_ concentrations during the day of enrollment. Moreover, very high correlation was observed among the three sources of exposure (R_Policlinico vs Average Milan_ = 0.99; R_Subjects’ Residence vs Average Milan_ = 0.94; R_Policlinico vs Subjects’ Residence_ = 0.99). We also calculated different lag time exposure windows from recruitment date using residential and Milan monitors.

**Figure 3:**
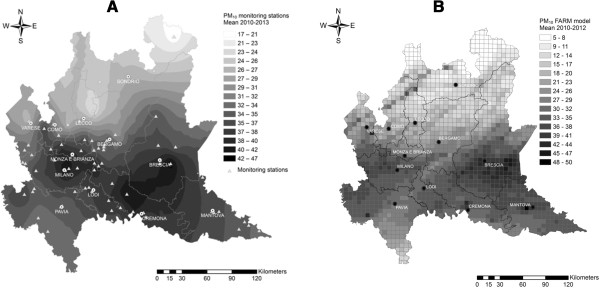
**Graphical representation of PM**_**10**_ **concentration levels.** **A**: Point measurements from monitoring stations expanded to the whole Lombardy territory through Empirical Bayesian Kriging (2010–2013). **B**: PM_10_ concentrations predicted by FARM model (2010–2012).

To give a map representation of PM_10_ from monitoring stations, we applied a geostatistical interpolated method (Empirical Bayesian kriging) that expands the monitor PM_10_ point observations to the whole Lombardy territory (Figure 3A) [62].

#### FARM model

Estimated daily average concentrations of PM_10_ for the years 2007–2012 based on the FARM model were obtained from ARPA Lombardy. The FARM model is a chemical transport model able to treat the main processes of chemical and physical nature involved in the formation and removal of pollutants, in addition to their transport and dispersion due to the action of wind and atmospheric mixing. The imputed data are built from meteorological observations of weather and hydrological network of ARPA and from processing of the results of the global meteorological model of the ECMWF (European Centre for Medium-Range Weather Forecasts). The initial and boundary conditions are obtained from the ARPA network data and the results of the model CHIMERE (http://www.lmd.polytechnique.fr/chimere/chimere.php). Finally, data on measured and simulated concentrations are harmonized through interpolation techniques [7, 63, 64]. By this model the Lombardy region is divided into a grid of 1678 cells (4×4 km), each associated with daily PM_10_ concentration estimates.

Hence, each subject was attributed the estimated mean daily exposure of: (1) the cell containing the residential address; (2) the cell containing the address of the Center for Obesity and Work; (3) the city of Milan, calculated as the average of the 22 cells that fall into the city boundaries.

Data estimated from the models are currently available until 2012, since the data validation imply a lag time of nearly 6 months, and will soon be available for further data analysis. A map of predicted PM_10_ concentrations (2010–2012) is shown in Figure 3B.

Both air quality measuring and modeling have some degree of uncertainty. Monitoring station measurements are often hampered by low spatial resolution (point data). On the other hand, modelling techniques have inherent constraints, due to the limited ability to perfectly describe physical phenomena and the intrinsic variability of the imputed data [65].

**Figure 4:**
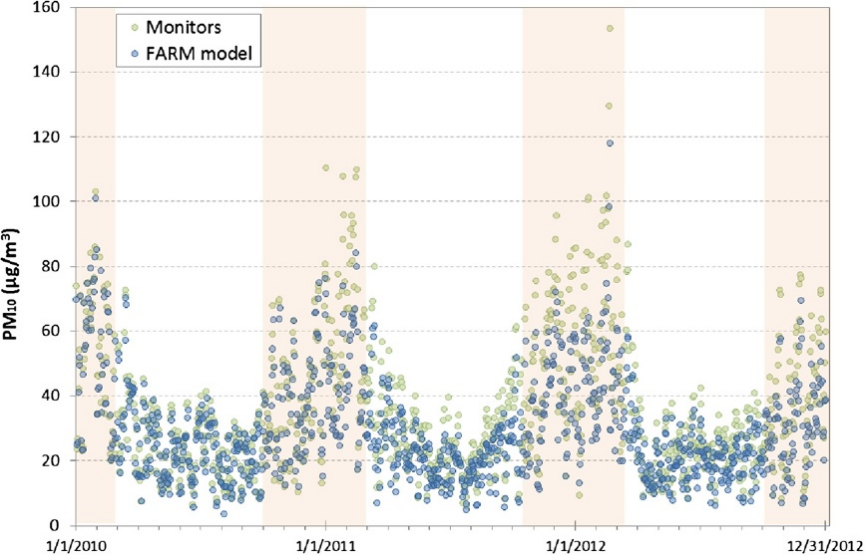
**Estimated and observed daily mean PM**_**10**_ **concentrations (2010–2012).** Daily mean PM10 concentrations of all monitors and of all grid cells with a monitor falling into their boundary. Darker area highlights winter months, characterized by major differences between the two methods of exposure assessment.

The distribution of daily mean PM_10_ concentrations (2010–2012) derived from both the monitoring stations and the FARM model is reported in Figure 4. PM_10_ estimates have, on average, lower values than measured concentrations (see also Additional file 1: Figure S1). During winter months (October to February) this difference is more evident (see also Additional file 2: Table S1).

FARM model performance was examined using a standard performance metric: the Mean Fractional Bias (MFB), which is a measure of the model tendency to over- or under-predict the observations from monitors. The MFB is defined as the normalized average difference between all model-observed pairs and can vary between ±200%. The model performance goal is met when the MFB is between ±30% [65, 66]. We obtained MFB = -16% for the entire period of comparison and year specific MFBs remained within the range. These results suggest the fulfillment of the objective of performance, confirm the tendency to underestimate the measured concentrations, and are consistent with the ARPA Lombardy Annual Assessment Of Air Quality Modeling for years 2009–2011 [64].

According to the Italian Legislative Decree 13 August, 2010, nr. 155 (Annex I), implementing the European Union Directive 2008/50/EC, ratios between measured and estimated data must be between ±50% in order to consider the data quality as acceptable [64, 67]. Our calculated ratios all fell in the data quality range, except for three monitors not linked to any subjects’ residence (see Additional file 1: Figure S2). Analyses performed for each year and over the entire time period gave comparable results.

In interpreting the above listed exposure assessment findings, it is important to consider that measurements from monitoring stations should not necessarily be considered as the gold-standard. The differences between the measured and estimated air pollutant levels may be partially explained by the fact that a point measurement is compared to a mean concentration modeled in volumetric grid cell. The former unlikely represents the average concentration over the same volume of air, making it difficult to obtain a perfect match between the two methods. The ability of the model to deeply take into account meteorological variables could also play an important role [65].

#### Internal dose of metals/elements

As part of the routine protocol, for each subject presenting at the center a 50 ml urine sample and a hair sample are collected, in order to quantify metals in urine and hair. Urine gives short term information (few hours before sample collection). Hair is also regarded as a valuable matrix as during its growth (about 1 cm per month) it accumulates toxic substances. We analyze a lock of hair (a 3 cm-length segment), cut next to the root in the occipital area of the head, roughly corresponding to 3 months of exposure. The parallel quantification of metals in urine and hair gives complementary data on medium- and short-term exposure. Trace metals/elements measured by ICP/MS include: Aluminum, Antimony, Arsenic, Beryllium, Bismuth, Cadmium, Lead, Mercury, Platinum, Thallium, Uranium, Nickel, Silver, Titanium, Calcium, Magnesium, Sodium, Potassium, Copper, Zinc, Manganese, Chromium, Vanadium, Molybdenum, Boron, Iodine, Lithium, Phosphorus, Selenium, Strontium, Sulfur, Barium, Cobalt, Iron, Germanium, Rubidium, Zirconium.

### Ethical issues

The study design, research aims and measurements have been approved by the local Institutional Review Board (‘Fondazione IRCCS Cà Granda Ospedale Maggiore Policlinico’ review board).

Each participant was asked to sign a written informed consent explaining the study in detail. New measurements will only be embedded in the study after approval of the Ethical Committee.

### Data management and privacy protection

A study collecting a large amount of information which are updated daily, such as SPHERE, requires highly efficient data management systems.

In order to protect the privacy of each subject, all information and biological samples collected from the subject are anonymized of personally identifying information and will be identified only through a 5-digit randomly assigned barcode. The link between the barcode and the subject’s identity is held in a secure database.

All questionnaire data collected through paper forms, are imputed in the database and quality and completeness control are performed weekly. All data are combined in a central relational database (MS SQL Server).

Data processing is anonymous and the highest level of confidentiality is maintained for all identifying information. We routinely check quality of collected data by comparing information from different sources (e.g. questionnaires, clinical records, biochemical exams); assessing variable range and distribution; evaluating the quality of biospecimens through specific analyses conducted on random samples; and verifying the database completeness through multiple queries.

A complete list of all collected variables is reported in Additional file 2: Table S2.

### Dissemination of results

A project’s website is being implemented and will be updated regularly (http://users.unimi.it/sphere). It will contain complete information about the project and relevant events. All publishable material and reports will be put online as they are produced. The results from the project will be converted into user-friendly protocols and published in press releases, educational programs, conferences, and scientific journals.

### Opportunities to collaborate

We designed SPHERE being aware that much of its value would arise from involvement with other investigators, individually and within consortia. The SPHERE project is open for collaboration with interested investigators. Given the detailed epidemiological data available, such as the clinical information collected, and the molecular data, SPHERE will provide a good environment for collaborations. Proposals from outside the study team for research projects to test specific hypotheses on SPHERE population will be reviewed by our research team. Requests can be sent to the e-mail sphere@unimi.it.

## Discussion

The investigation of mechanisms linking air pollutant inhalation to cardiovascular effects is being considered a pressing priority [40, 68].

The SPHERE study is, at the best of our knowledge, the first study primarily designed to determine whether exposure to air particles and PM-associated metals can modify EVs (as quantity, size, membrane molecules, procoagulant activity and miRNA content) in plasma of human subjects and to investigate whether these alterations may be linked to cardiovascular risk factors and outcomes.

The identification of miRNAs in plasma EVs of healthy subjects [47] provides the basis for a new potential mechanism, since EVs produced by lung or dendritic cells might be able to transfer a specific pattern of miRNAs to immune and endothelial cells.

We will be able to examine the above mentioned mechanism in a large group of human subjects particularly susceptible to the effects of air pollution, measuring EV production and content (miRNAs) in plasma. For this purpose, we developed a novel methodology which involves the characterization of microvesicular membrane determinants (to assess their cellular origin) by Flow cytometry, microvesicle sizing and counting by Nanosight technology, and microvesicle content (miRNAs) analysis by highly quantitative OpenArray technology (Quant Studio, Applied Bioscience). Openarray technique will allow to analyze, in an unbiased way, the entire miRNome in one single reaction and will produce very precise and accurate data. Moreover, the inclusion in the study protocol of a discovery stage (1000 samples) followed by a confirmation stage (1000 samples), will lower the possibility to find false positive results.

Thus, if we were successful in identifying miRNA alterations in EVs, we could detect the opportunity of using an easily obtainable biological media (plasma) for potential future preventive and diagnostic applications. In addition, as miRNAs in EVs may be a possible drug target, our study might also open paths to develop future interventions to reverse the effects of air pollution.

The extensive clinical and biochemical data collected for each subject will help in examining the relationships under study taking into account the possible role of other covariates.

The characterization of subjects’ exposures can rely on various sources of exposure assessment: monitoring stations, personal samplers, model estimates, and internal dose measurements.

The availability of PM measurements from past years and the use of diverse lag-time windows will allow to investigate both long- and short-term exposure effects. Residential, workplace and mobility information collected through questionnaires will improve the accuracy of subject-specific exposure assessment.

A further validation of the exposure indexes will be provided by the use of passive samplers in a subgroup of subjects. We will also integrate all the information with urine and hair metal measurements.

Finally, the huge amount of available data will offer the opportunity to implement further new modelling techniques as long as they might come out.

In conclusion, SPHERE is the first large study aimed to explore EVs as a novel potential mechanism of action of air pollution exposure in a highly susceptible population. The rigorous study design, the availability of banked biological samples and the potential to integrate epidemiological, clinical and molecular data will also furnish a powerful base for investigating different complementary molecular mechanisms.

## Electronic supplementary material

Additional file 1: Figure S1: Estimated and observed mean PM_10_ level by place. **Figure S2.** Data quality control on FARM model estimates (2010–2012) according to EU Directive 2008/50/EC. Each point represents PM_10_ mean concentrations as measured by monitoring stations (x axis) and estimated by FARM model in the corresponding cells (y axis). The cone dotted lines delimit the ±50% range of data quality. (DOCX 354 KB)

Additional file 2: Table S1: PM_10_ profile (overall and by season) and selected weather variables (2010–2012). All values are calculated at the time of blood sampling. **Table S2.** Complete list of variables collected for the SPHERE study. (DOCX 36 KB)

## Abbreviations

BMI: Body Mass Index
CTM: Chemical Transport Models
ECG: Electrocardiogram
EVs: Extracellular vesicles
FEF_25_: Forced expiratory flows at 25%
FEF_50_: Forced expiratory flows at 50%
FEF_75_: Forced expiratory flows at 75%
FEV_1_: Forced expiratory volume in one second
FVC: Forced vital capacity
miRNAs: microRNAs
PEF: Peak expiratory flow
PM: Particulate Matter.
