# Supplementary material for: Susceptibility to particle health effects, miRNA and exosomes: rationale and study protocol of the SPHERE study
Source: BMC Public Health. 2014 Nov 4;14:1137. doi: 10.1186/1471-2458-14-1137 (PMC4242553; doi:10.1186/1471-2458-14-1137)
Supplement: Supplementary file 1 — Additional file 1: Figure S1: Estimated and observed mean PM10 level by place. Figure S2. Data quality control on FARM model estimates (2010–2012) according to EU Directive 2008/50/EC. Each point represents PM10 mean concentrations as measured by monitoring stations (x axis) and estimated by FARM model in the corresponding cells (y axis). The cone dotted lines delimit the ±50% range of data quality. (DOCX 354 KB) [file 12889_2014_7251_MOESM1_ESM.docx]

**Supplementary Figure 1:** Estimated and observed mean PM_10_ level by place.


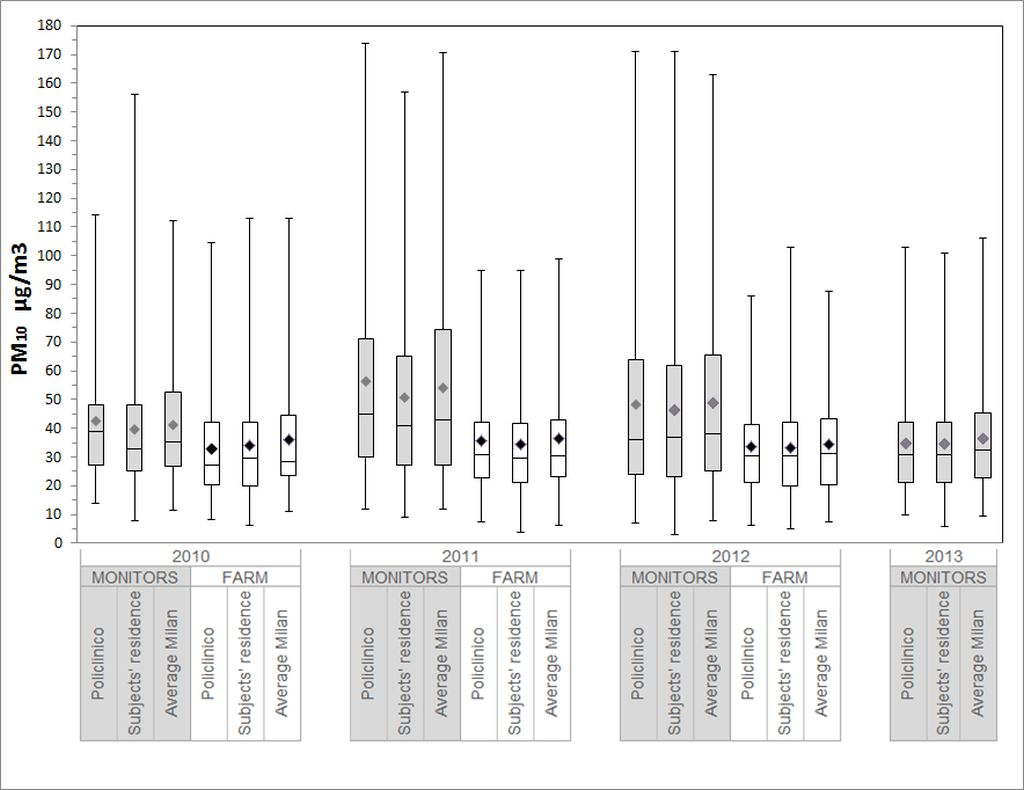


**Supplementary Figure 2:** Data quality control on FARM model estimates (2010-2012) according to EU Directive 2008/50/EC.


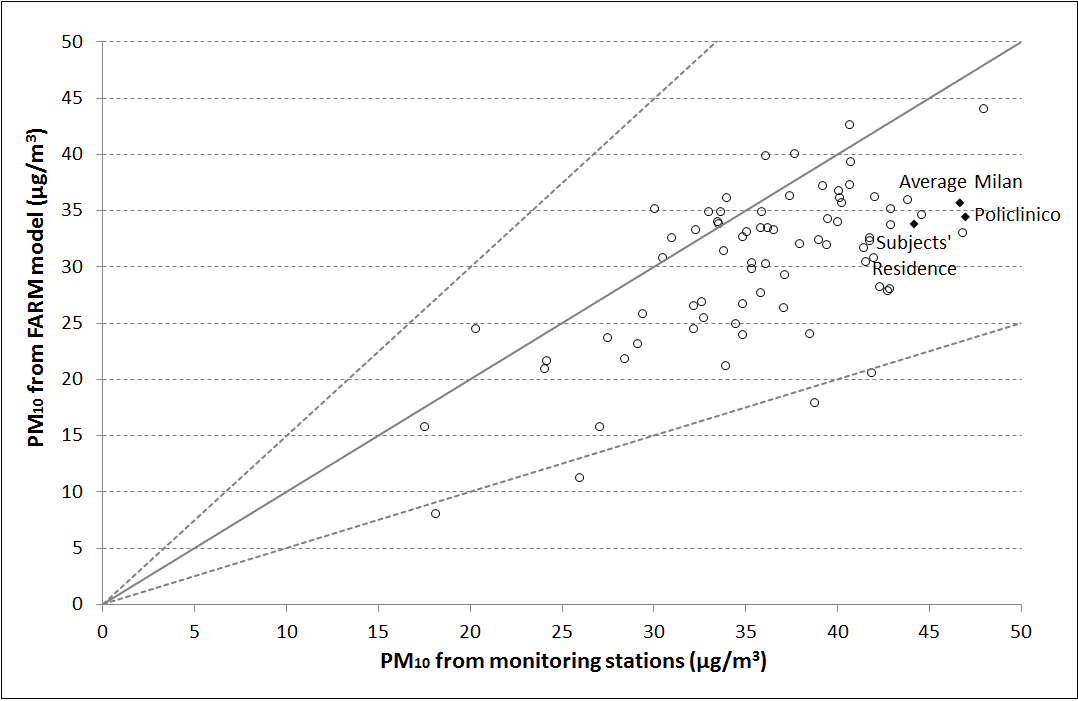


Each point represents PM_10_ mean concentrations as measured by monitoring stations (x axis) and estimated by FARM model in the corresponding cells (y axis). The cone dotted lines delimit the ±50% range of data quality.
