# Supplementary material for: Susceptibility to particle health effects, miRNA and exosomes: rationale and study protocol of the SPHERE study
Source: BMC Public Health. 2014 Nov 4;14:1137. doi: 10.1186/1471-2458-14-1137 (PMC4242553; doi:10.1186/1471-2458-14-1137)
Supplement: Supplementary file 2 — Additional file 2: Table S1: PM10 profile (overall and by season) and selected weather variables (2010–2012). All values are calculated at the time of blood sampling. Table S2. Complete list of variables collected for the SPHERE study. (DOCX 36 KB) [file 12889_2014_7251_MOESM2_ESM.docx]

**Supplementary Table 1.** PM_10_ profile (overall and by season) and selected weather variables (2010-2012). All values are calculated at the time of blood sampling.

|  | |  | | **Mean** | | **SD** | | **Min** | | | **Q1** | | | **Median** | | | | **Q3** | | **Max** | | |  |
| --- | --- | --- | --- | --- | --- | --- | --- | --- | --- | --- | --- | --- | --- | --- | --- | --- | --- | --- | --- | --- | --- | --- | --- |
| **PM_10_ (µg/m^3^)** | | | |  | |  | |  | | |  | | |  | | | |  | |  | | |  |
|  | |  | |  | |  | |  | | |  | | |  | | | |  | |  | | |  |
| *Monitory station (n=1250)* | | | |  | |  | |  | | |  | | |  | | | |  | |  | | |  |
|  | | Policlinico | | 47.0 | | 30.5 | | 7.0 | | | 26.0 | | | 38.0 | | | | 59.0 | | 174.0 | | |  |
|  | | Subjects’ residence | | 44.2 | | 28.3 | | 3.0 | | | 24.0 | | | 36.0 | | | | 56.0 | | 171.0 | | |  |
|  | | Average Milan | | 46.7 | | 29.5 | | 7.7 | | | 25.7 | | | 37.7 | | | | 60.0 | | 170.7 | | |  |
| *Eulerian Model Estimate (n=931)* | | | |  | |  | |  | | |  | | |  | | | |  | |  | | |  |
|  | | Policlinico | | 34.5 | | 18.0 | | 6.2 | | | 21.6 | | | 29.8 | | | | 41.8 | | 104.7 | | |  |
|  | | Subjects’ residence | | 33.8 | | 18.3 | | 4.0 | | | 20.4 | | | 29.7 | | | | 42.1 | | 113.1 | | |  |
|  | | Average Milan | | 35.6 | | 18.7 | | 6.4 | | | 22.8 | | | 30.4 | | | | 43.1 | | 113.0 | | |  |
|  | |  | |  | |  | |  | | |  | | |  | | | |  | |  | | |  |
| ***Autumn/Winter*** | | | | |  | | |  | | |  | | |  |  | | | | |  | | |  |
| *Monitory station (n=747)* | | | | |  | | |  | | |  | | |  |  | | | | |  | | |  |
|  | | Policlinico | 58.8 | 33.5 | | 7.0 | | | 33.0 | | | 52.0 | | | | 76.0 | | 174.0 | | |  |  |  |
|  | | Subjects’ residence | 55.0 | 30.8 | | 3.0 | | | 33.0 | | | 48.0 | | | | 72.0 | | 171.0 | | |  |  |  |
|  | | Average Milan | 58.6 | 31.8 | | 11.3 | | | 34.3 | | | 51.3 | | | | 76.7 | | 170.7 | | |  |  |  |
| *Eulerian Model Estimate (n=569)* | | |  |  | |  | | |  | | |  | | | |  | |  | | |  |  |  |
|  | | Policlinico | 40.1 | 19.4 | | 7.4 | | | 26.6 | | | 35.9 | | | | 52.8 | | 104.7 | | |  |  |  |
|  | | Subjects’ residence | 39.5 | 19.6 | | 4.9 | | | 25.8 | | | 35.6 | | | | 51.4 | | 113.1 | | |  |  |  |
|  | | Average Milan | 41.7 | 20.0 | | 6.4 | | | 27.2 | | | 37.0 | | | | 55.3 | | 113.0 | | |  |  |  |
|  | |  |  |  | |  | | |  | | |  | | | |  | |  | | |  |  |  |
| ***Spring/Summer*** | | |  |  | |  | | |  | | |  | | | |  | |  | | |  |  |  |
| *Monitory station (n=503)* | | |  |  | |  | | |  | | |  | | | |  | |  | | |  |  |  |
|  | | Policlinico | 29.4 | 11.7 | | 8.0 | | | 22.0 | | | 27.0 | | | | 37.0 | | 85.0 | | |  |  |  |
|  | | Subjects’ residence | 28.2 | 12.2 | | 6.0 | | | 20.0 | | | 26.0 | | | | 35.0 | | 85.0 | | |  |  |  |
|  | | Average Milan | 28.9 | 11.8 | | 7.7 | | | 21.0 | | | 26.7 | | | | 36.3 | | 74.7 | | |  |  |  |
| *Eulerian Model Estimate (n=362)* | | |  |  | |  | | |  | | |  | | | |  | |  | | |  |  |  |
|  | | Policlinico | 25.6 | 10.7 | | 6.2 | | | 18.6 | | | 23.9 | | | | 30.6 | | 74.1 | | |  |  |  |
|  | | Subjects’ residence | 24.9 | 11.2 | | 4.0 | | | 17.6 | | | 22.3 | | | | 30.9 | | 69.2 | | |  |  |  |
|  | | Average Milan | 26.0 | 10.8 | | 7.2 | | | 18.8 | | | 24.2 | | | | 30.8 | | 73.1 | | |  |  |  |
| **Weather variables (all seasons) (n=1250)** | | | |  | |  | |  | | |  | | |  | | | |  | |  | | |  |
|  | | Relative humidity (%) | | 69.3 | | 18.4 | | 9.0 | | | 56.0 | | | 68.0 | | | | 85.0 | | 106.2 | | |  |
|  | | Temperature (°C) | | 12.6 | | 7.8 | | -8.0 | | | 6.3 | | | 12.1 | | | | 18.6 | | 30.0 | | |  |
|  | | Apparent temperature (°C) | | 11.2 | | 8.8 | | -9.0 | | | 3.8 | | | 10.6 | | | | 18.0 | | 31.9 | | |  |

SD: standard deviation, PM: particulate matter, Min: minimum, Q1: first quartile, Q3: third quartile, Max=maximum

**Supplementary table 2:** Complete list of variables collected for the SPHERE study.

| **Variable name** | **Variable classification** | **Original Source** |
| --- | --- | --- |
| Birth Date | Socio-demographic | Lifestyle Questionnaire (verified by Clinical records) |
| Visit and blood drawing date | Socio-demographic | Lifestyle Questionnaire (verified by Clinical records) |
| Sex | Socio-demographic | Lifestyle Questionnaire (verified by Clinical records) |
| Education | Socio-demographic | Lifestyle Questionnaire |
| Working status | Socio-demographic | Lifestyle Questionnaire |
| If retired, year of retirement | Socio-demographic | Lifestyle Questionnaire |
| If working, occupation | Socio-demographic | Lifestyle Questionnaire |
| Working address | Socio-demographic | Lifestyle Questionnaire |
| Home address | Socio-demographic | Lifestyle Questionnaire |
|  |  |  |
| Pregnancy number | Reproductive | Clinical records |
| Miscarriages number | Reproductive | Clinical records |
| Live birth number | Reproductive | Clinical records |
| Menopause (Y/N) | Reproductive | Clinical records |
|  |  |  |
| Waist circumference | Obesity-related parameters | Clinical records |
| Neck circumference | Obesity-related parameters | Clinical records |
| Waist to Hip Ratio (WHR) | Obesity-related parameters | Clinical records |
| Homeostatic Model Assessment (HOMA) index | Obesity-related parameters | Clinical records |
| Body Fat percentage | Obesity-related parameters | Clinical records |
| Estimated Basal Metabolism | Obesity-related parameters | Clinical records |
| Obesity Class | Obesity-related parameters | Clinical records |
| Vascular Risk at 10 years | Obesity-related parameters | Clinical records |
|  |  |  |
| Insulin Resistance (Y/N) | Pathologic Condition | Lifestyle Questionnaire (verified by Clinical records) |
| Metabolic Syndrome (Y/N) | Pathologic Condition | Lifestyle Questionnaire (verified by Clinical records) |
| Dyslipidemia (Y/N) | Pathologic Condition | Lifestyle Questionnaire (verified by Clinical records) |
| Diabetes (Y/N) | Pathologic Condition | Lifestyle Questionnaire (verified by Clinical records) |
| Hypertension (Y/N) | Pathologic Condition | Lifestyle Questionnaire (verified by Clinical records) |
| Inherited Metabolic Diseases (Y/N) | Pathologic Condition | Lifestyle Questionnaire (verified by Clinical records) |
| Hepatic Failure (Y/N) | Pathologic Condition | Lifestyle Questionnaire (verified by Clinical records) |
| Pancreatic Failure (Y/N) | Pathologic Condition | Lifestyle Questionnaire (verified by Clinical records) |
| Renal Failure (Y/N) | Pathologic Condition | Lifestyle Questionnaire (verified by Clinical records) |
| Celiac Disease (Y/N) | Pathologic Condition | Lifestyle Questionnaire (verified by Clinical records) |
| Severe food intolerances (Y/N) | Pathologic Condition | Lifestyle Questionnaire (verified by Clinical records) |
| Cancer (Y/N) | Pathologic Condition | Lifestyle Questionnaire (verified by Clinical records) |
| Stroke (Y/N) | Pathologic Condition | Lifestyle Questionnaire (verified by Clinical records) |
| Osteoporosis (Y/N) | Pathologic Condition | Lifestyle Questionnaire (verified by Clinical records) |
| Colon Cancer | Pathologic Condition | Lifestyle Questionnaire (verified by Clinical records) |
| Lung Cancer | Pathologic Condition | Lifestyle Questionnaire (verified by Clinical records) |
| Breast Cancer | Pathologic Condition | Lifestyle Questionnaire (verified by Clinical records) |
| Uterine Cancer | Pathologic Condition | Lifestyle Questionnaire (verified by Clinical records) |
| First grade relative with Diabetes | Pathologic Condition | Lifestyle Questionnaire (verified by Clinical records) |
| First grade relative with Cardiovascular Disease | Pathologic Condition | Lifestyle Questionnaire (verified by Clinical records) |
| Melanoma | Pathologic Condition | Lifestyle Questionnaire (verified by Clinical records) |
| Other malignant cancer | Pathologic Condition | Lifestyle Questionnaire (verified by Clinical records) |
|  |  |  |
| Medications (regularly assumed) | Lifestyle factors | Lifestyle Questionnaire (verified by Clinical records) |
| Medications (recently assumed) | Lifestyle factors | Lifestyle Questionnaire (verified by Clinical records) |
| Area of Residence | Lifestyle factors | Lifestyle Questionnaire |
| Floor of Residence | Lifestyle factors | Lifestyle Questionnaire |
| Residence traffic exposure | Lifestyle factors | Lifestyle Questionnaire |
| Commuting time | Lifestyle factors | Lifestyle Questionnaire |
| Transport mode | Lifestyle factors | Lifestyle Questionnaire |
| Smoking status | Lifestyle factors | Lifestyle Questionnaire |
| If current smoker, number of cigarettes | Lifestyle factors | Lifestyle Questionnaire |
| If current/former smoker, starting age | Lifestyle factors | Lifestyle Questionnaire |
| If former smoker, quitting age | Lifestyle factors | Lifestyle Questionnaire |
| Pack/Years | Lifestyle factors | Lifestyle Questionnaire |
| Passive smoking | Lifestyle factors | Lifestyle Questionnaire |
| Physical Activity | Lifestyle factors | Lifestyle Questionnaire |
| Frequency of physical activity | Lifestyle factors | Lifestyle Questionnaire |
| Time spent at PC/TV | Lifestyle factors | Lifestyle Questionnaire |
|  |  |  |
| Systolic Blood Pressure | Cardiovascular Function | Clinical records |
| Diastolic Blood Pressure | Cardiovascular Function | Clinical records |
| Heart Rate | Cardiovascular Function | Clinical records (from ECG) |
| P-axis | Cardiovascular Function | Clinical records (from ECG) |
| QRS-axis | Cardiovascular Function | Clinical records (from ECG) |
| T-axis | Cardiovascular Function | Clinical records (from ECG) |
| P-R interval | Cardiovascular Function | Clinical records (from ECG) |
| QRS duration | Cardiovascular Function | Clinical records (from ECG) |
| QT interval | Cardiovascular Function | Clinical records (from ECG) |
| Corrected QT (QTc) | Cardiovascular Function | Clinical records (from ECG) |
|  |  |  |
| Uric acid | Biochemical test | Laboratory of Clinical Chemistry and Microbiology |
| Fibrinogen | Biochemical test | Laboratory of Clinical Chemistry and Microbiology |
| C-reactive protein | Biochemical test | Laboratory of Clinical Chemistry and Microbiology |
| Total cholesterol | Biochemical test | Laboratory of Clinical Chemistry and Microbiology |
| HDL | Biochemical test | Laboratory of Clinical Chemistry and Microbiology |
| LDL | Biochemical test | Laboratory of Clinical Chemistry and Microbiology |
| Triglyceride | Biochemical test | Laboratory of Clinical Chemistry and Microbiology |
| Serum creatinine | Biochemical test | Laboratory of Clinical Chemistry and Microbiology |
| AST, U/I | Biochemical test | Laboratory of Clinical Chemistry and Microbiology |
| ALT, U/I | Biochemical test | Laboratory of Clinical Chemistry and Microbiology |
| Gamma-Glutamyltransferase | Biochemical test | Laboratory of Clinical Chemistry and Microbiology |
| Glucose | Biochemical test | Laboratory of Clinical Chemistry and Microbiology |
| Homocysteine | Biochemical test | Laboratory of Clinical Chemistry and Microbiology |
| TSH | Biochemical test | Laboratory of Clinical Chemistry and Microbiology |
| Glycated hemoglobin | Biochemical test | Laboratory of Clinical Chemistry and Microbiology |
| Postprandial glycaemia | Biochemical test | Laboratory of Clinical Chemistry and Microbiology |
| Insulin level | Biochemical test | Laboratory of Clinical Chemistry and Microbiology |
| 2-hour post glucose insulin level | Biochemical test | Laboratory of Clinical Chemistry and Microbiology |
| Vitamine D | Biochemical test | Laboratory of Clinical Chemistry and Microbiology |
| Urinary pH | Biochemical test | Laboratory of Clinical Chemistry and Microbiology |
| White blood cell count | Biochemical test | Laboratory of Clinical Chemistry and Microbiology |
| Red blood cells count | Biochemical test | Laboratory of Clinical Chemistry and Microbiology |
| Hemoglobin | Biochemical test | Laboratory of Clinical Chemistry and Microbiology |
| Hematocrit | Biochemical test | Laboratory of Clinical Chemistry and Microbiology |
| Mean Corpuscolar Volume | Biochemical test | Laboratory of Clinical Chemistry and Microbiology |
| Platelets | Biochemical test | Laboratory of Clinical Chemistry and Microbiology |
| Neutrophils count | Biochemical test | Laboratory of Clinical Chemistry and Microbiology |
| Neutrophils % | Biochemical test | Laboratory of Clinical Chemistry and Microbiology |
| Eosinophils count | Biochemical test | Laboratory of Clinical Chemistry and Microbiology |
| Eosinophils % | Biochemical test | Laboratory of Clinical Chemistry and Microbiology |
| Lymphocytes count | Biochemical test | Laboratory of Clinical Chemistry and Microbiology |
| Lymphocytes % | Biochemical test | Laboratory of Clinical Chemistry and Microbiology |
| Monocytes count | Biochemical test | Laboratory of Clinical Chemistry and Microbiology |
| Monocytes % | Biochemical test | Laboratory of Clinical Chemistry and Microbiology |
| Basophil count | Biochemical test | Laboratory of Clinical Chemistry and Microbiology |
| Basophil % | Biochemical test | Laboratory of Clinical Chemistry and Microbiology |
| Granulocytes count | Biochemical test | Laboratory of Clinical Chemistry and Microbiology |
| Granulocytes % | Biochemical test | Laboratory of Clinical Chemistry and Microbiology |
|  |  |  |
| Forced vital capacity (FVC) | Respiratory function | Clinical records (from spirometry) |
| Forced expiratory volume in 1 second (FEV1) | Respiratory function | Clinical records (from spirometry) |
| FEV1/FVC ratio (FEV1%) | Respiratory function | Clinical records (from spirometry) |
| Forced expiratory flow (FEF) | Respiratory function | Clinical records (from spirometry) |
| Forced inspiratory flow 25–75% or 25–50% | Respiratory function | Clinical records (from spirometry) |
| Peak expiratory flow (PEF) | Respiratory function | Clinical records (from spirometry) |
| Total lung capacity (TLC) | Respiratory function | Clinical records (from spirometry) |
| Diffusing capacity (DLCO) | Respiratory function | Clinical records (from spirometry) |
| Inspiratory vital capacity (IVC) | Respiratory function | Clinical records (from spirometry) |
| Maximal Expiratory Flow (MEF) | Respiratory function | Clinical records (from spirometry) |
| MEF at 25% of Vital Flow Capacity (MEF25) | Respiratory function | Clinical records (from spirometry) |
| MEF at 50% of Vital Flow Capacity (MEF50) | Respiratory function | Clinical records (from spirometry) |
| MEF at 75% of Vital Flow Capacity (MEF75) | Respiratory function | Clinical records (from spirometry) |
| Residual volume (RV) | Respiratory function | Clinical records (from spirometry) |
|  |  |  |
| Egg Stuffed Pasta | Weekly/Monthly diet | Diet Questionnaire |
| Pasta, Rice | Weekly/Monthly diet | Diet Questionnaire |
| Soup With Pasta | Weekly/Monthly diet | Diet Questionnaire |
| Bread | Weekly/Monthly diet | Diet Questionnaire |
| Polenta | Weekly/Monthly diet | Diet Questionnaire |
| Pizza | Weekly/Monthly diet | Diet Questionnaire |
| Bread Whole | Weekly/Monthly diet | Diet Questionnaire |
| Crackers, Rusks, Bread Sticks | Weekly/Monthly diet | Diet Questionnaire |
| Cookies, Brioches | Weekly/Monthly diet | Diet Questionnaire |
| Cornflakes | Weekly/Monthly diet | Diet Questionnaire |
| Snacks | Weekly/Monthly diet | Diet Questionnaire |
| Pizza-Snack, White Pizza Bun | Weekly/Monthly diet | Diet Questionnaire |
| Beans, Lentils, Chickpeas, Broad Beans, Soy | Weekly/Monthly diet | Diet Questionnaire |
| Fresh Cooked Peas | Weekly/Monthly diet | Diet Questionnaire |
| Potatoes | Weekly/Monthly diet | Diet Questionnaire |
| Carrots | Weekly/Monthly diet | Diet Questionnaire |
| Raw Tomatoes | Weekly/Monthly diet | Diet Questionnaire |
| Tomato Sauce; Tomato Puree | Weekly/Monthly diet | Diet Questionnaire |
| Salad Leaf | Weekly/Monthly diet | Diet Questionnaire |
| Cauliflower, Sprouts, Cabbage, Broccoli | Weekly/Monthly diet | Diet Questionnaire |
| Spinach, Beets, Chard, Herbs And Catalonia | Weekly/Monthly diet | Diet Questionnaire |
| Peppers | Weekly/Monthly diet | Diet Questionnaire |
| Zucchini, Green Beans, Eggplant, Artichokes, Cucumbers | Weekly/Monthly diet | Diet Questionnaire |
| Citrus | Weekly/Monthly diet | Diet Questionnaire |
| Apples | Weekly/Monthly diet | Diet Questionnaire |
| Apricot, Bananas, Pears, Peaches, Plums, Grapes | Weekly/Monthly diet | Diet Questionnaire |
| Nuts | Weekly/Monthly diet | Diet Questionnaire |
| Melon, Watermelon | Weekly/Monthly diet | Diet Questionnaire |
| Olive Oil | Weekly/Monthly diet | Diet Questionnaire |
| Seed Oil | Weekly/Monthly diet | Diet Questionnaire |
| Butter | Weekly/Monthly diet | Diet Questionnaire |
| Mayonnaise | Weekly/Monthly diet | Diet Questionnaire |
| Beef White Or Red | Weekly/Monthly diet | Diet Questionnaire |
| Chicken, Turkey, Rabbit | Weekly/Monthly diet | Diet Questionnaire |
| Pork | Weekly/Monthly diet | Diet Questionnaire |
| Raw Ham | Weekly/Monthly diet | Diet Questionnaire |
| Baked Ham | Weekly/Monthly diet | Diet Questionnaire |
| Bologna, Sausage | Weekly/Monthly diet | Diet Questionnaire |
| Salami, “Coppa” | Weekly/Monthly diet | Diet Questionnaire |
| Whole Milk, Whole Yogurt | Weekly/Monthly diet | Diet Questionnaire |
| Semi-Skimmed Milk, Low-Fat Yogurt | Weekly/Monthly diet | Diet Questionnaire |
| Green Cheese | Weekly/Monthly diet | Diet Questionnaire |
| Ripened Cheese | Weekly/Monthly diet | Diet Questionnaire |
| Parmesan Cheese | Weekly/Monthly diet | Diet Questionnaire |
| Grated Parmesan Cheese | Weekly/Monthly diet | Diet Questionnaire |
| Eggs | Weekly/Monthly diet | Diet Questionnaire |
| Fresh Or Frozen Fish | Weekly/Monthly diet | Diet Questionnaire |
| Tuna, Other Fish In Oil | Weekly/Monthly diet | Diet Questionnaire |
| Chocolate | Weekly/Monthly diet | Diet Questionnaire |
| Crisp | Weekly/Monthly diet | Diet Questionnaire |
| Icecream | Weekly/Monthly diet | Diet Questionnaire |
| Sweet Sparkling Drinks | Weekly/Monthly diet | Diet Questionnaire |
| Fruit Juice (All Types) | Weekly/Monthly diet | Diet Questionnaire |
| Honey | Weekly/Monthly diet | Diet Questionnaire |
| Wine (Glasses) | Weekly/Monthly diet | Diet Questionnaire |
| Beer (Jugs) | Weekly/Monthly diet | Diet Questionnaire |
| Hard Liquor | Weekly/Monthly diet | Diet Questionnaire |
| Coffee (Small Cups) | Weekly/Monthly diet | Diet Questionnaire |
| The (Cups) | Weekly/Monthly diet | Diet Questionnaire |
|  |  |  |
| Saturated fat | Estimated dietary intake | Calculated from Diet Questionnaire |
| Cholesterol | Estimated dietary intake | Calculated from Diet Questionnaire |
| Retinol | Estimated dietary intake | Calculated from Diet Questionnaire |
| Tocopherols | Estimated dietary intake | Calculated from Diet Questionnaire |
| Omega 3 | Estimated dietary intake | Calculated from Diet Questionnaire |
| Fiber | Estimated dietary intake | Calculated from Diet Questionnaire |
| Vitamin B12 | Estimated dietary intake | Calculated from Diet Questionnaire |
| Carbohydrates | Estimated dietary intake | Calculated from Diet Questionnaire |
| Calcium | Estimated dietary intake | Calculated from Diet Questionnaire |
| Alcohol | Estimated dietary intake | Calculated from Diet Questionnaire |
| Folic Acid | Estimated dietary intake | Calculated from Diet Questionnaire |
| Vitamin D | Estimated dietary intake | Calculated from Diet Questionnaire |
| Ascorbic Acid | Estimated dietary intake | Calculated from Diet Questionnaire |
| Protein | Estimated dietary intake | Calculated from Diet Questionnaire |
| Lipids | Estimated dietary intake | Calculated from Diet Questionnaire |
| Carotenoids | Estimated dietary intake | Calculated from Diet Questionnaire |
| Iron | Estimated dietary intake | Calculated from Diet Questionnaire |
| Zinc | Estimated dietary intake | Calculated from Diet Questionnaire |
| Beta Carotene | Estimated dietary intake | Calculated from Diet Questionnaire |
| Crypto Xanthine | Estimated dietary intake | Calculated from Diet Questionnaire |
| Polyphenols, Flavonoids | Estimated dietary intake | Calculated from Diet Questionnaire |
| Monounsaturated Fat | Estimated dietary intake | Calculated from Diet Questionnaire |
| Lycopene | Estimated dietary intake | Calculated from Diet Questionnaire |
| Alpha Carotene | Estimated dietary intake | Calculated from Diet Questionnaire |
| Polyunsaturated Fatty | Estimated dietary intake | Calculated from Diet Questionnaire |
| Lutein, Zeaxanthin | Estimated dietary intake | Calculated from Diet Questionnaire |
| Starch | Estimated dietary intake | Calculated from Diet Questionnaire |
|  |  |  |
